# Supplementary material for: Diurnal Variation in and Optimal Time to Measure Holter-Based Late Potentials to Predict Lethal Arrhythmia after Myocardial Infarction
Source: Medicina (Kaunas). 2023 Aug 13;59(8):1460. doi: 10.3390/medicina59081460 (PMC10456944; doi:10.3390/medicina59081460)
Supplement: Supplementary file 1 [file medicina-59-01460-s001.zip › medicina-2457696-supplementary.pdf]

Supplementary Materials: Diurnal Variation in and Optimal Time to Measure Holter-Based Late Potentials to Predict Lethal Arrhythmia after Myocardial Infarction

Kenichi Hashimoto*, Naomi Harada, Motohiro Kimata, Yusuke Kawamura, Naoya Fujita, Akinori Sekizawa, Yosuke Ono, Yasuhiro Obuchi, Tadateru Takayama, Yuji Kasamaki and Yuji Tanaka

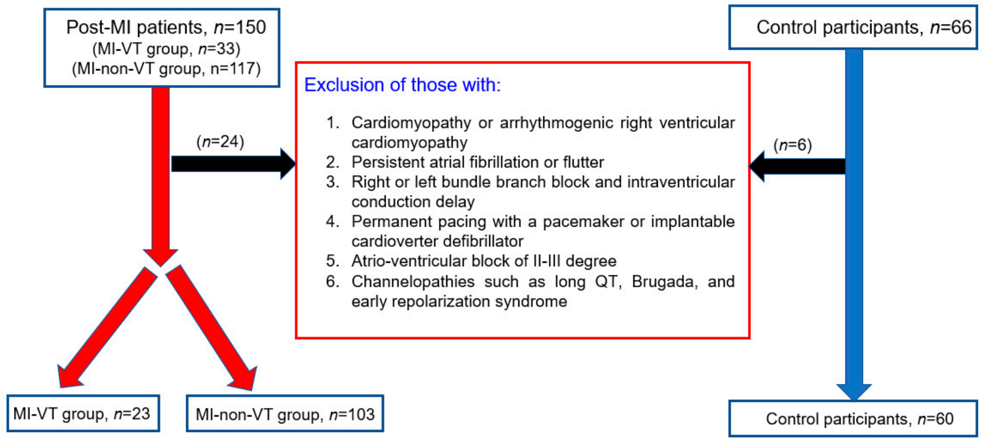

Figure S1. Study population.
